# Mammary-type myofibroblastoma of the right thigh: a case report and review of the literature

**DOI:** 10.1186/s13256-015-0601-0

**Published:** 2015-06-02

**Authors:** Jamshid Abdul-Ghafar, Nasir Ud Din, Zubair Ahmad, Steven D Billings

**Affiliations:** 1 Department of Pathology and Laboratory Medicine, Aga Khan University Hospital, Stadium Road, P.O. Box 3500 Karachi, Pakistan; 2 Department of Laboratory Medicine, Pathology Section, French Medical Institute for Children, behind Kabul Medical University Aliabad, P.O. Box: 472 Kabul, Afghanistan; 3 Department of Pathology, Cleveland Clinic, 9500 Euclid Ave, Cleveland, OH 44195 USA

**Keywords:** Immunohistochemistry, Myofibroblastoma, Mammary-type, Thigh

## Abstract

**Introduction:**

Mammary-type myofibroblastoma of the soft tissue is a very rare, benign, mesenchymal neoplasm with myofibroblastic differentiation. To date, 20 cases of extra-mammary myofibroblastoma have been described in literature. To the best of our knowledge, this is the largest extra-mammary myofibroblastoma described in the literature, and the first case reported in this location.

**Case presentation:**

A 50-year-old Pakistani man presented with a long history of a painless, huge lump on his right thigh. His clinical examination showed normal-looking skin and there was no inguinal lymphadenopathy. The mass was excised with a clinical impression of soft tissue sarcoma. Gross examination showed a huge, well-circumscribed soft tissue mass measuring 34cm in its largest dimension and weighing approximately 13kg. It was partially covered by fat tissue. Histologically, the lesion was composed of a haphazard arrangement of bland spindle-shaped cell fascicles in a thick collagenous and myxoid background. The neoplastic cells showed diffuse and patchy positivity for CD34 and desmin, respectively. No recurrence was seen following surgical excision over a follow-up period of five months.

**Conclusions:**

Mammary-type myofibroblastoma of the soft tissue is a benign soft tissue neoplasm, and no malignant behavior and/or recurrence after surgical resection has been described, regarding its size and location. As an extremely rare tumor, the correct diagnosis and prompt management is important, and requires careful clinical and pathological workup to rule out the possibility of a malignant neoplasm.

## Introduction

Mammary-type myofibroblastoma is a rare, benign, soft tissue tumor which morphologically resembles its mammary counterpart. The lesion was first described in 1987 by Wargotz *et al*. [1] as a distinctive, benign, mesenchymal tumor of the breast. The lesion is usually well-circumscribed, composed of haphazardly arranged fascicles of spindle-shaped cells admixed with interrupted adipocytes in a collagenous and myxoid background. The presence of coffee-bean shaped nuclear grooves, which was first described by Wargotz *et al*. [1], is a cytological clue for diagnosis in fine-needle aspiration cytology smears [2]. The most common immunohistochemical profile seen in these tumors is diffuse positivity for CD34 and desmin [3]; however, more variable positivity has also been reported [4]. This tumor is most often confused with spindle cell lipoma, as both are spindle cell neoplasms, both are positive for CD34, and in addition at the molecular level, both reveal similar loss of genetic material from the 13q14 region [3,5]. The standard management is surgical removal. Malignant behavior or recurrence has not been described.

A review of the literature revealed a total 20 cases of extra-mammary myofibroblastoma described to date, with a slightly male predilection [3,5-13]. The largest size reported in the literature was 13cm in diameter, and the most common location was the inguinal area (Table 1) [3]. Here we report a case of mammary-type myofibroblastoma, which we believe is the largest case (34cm in largest diameter) described so far, in the right thigh of a 50-year-old man. The literature is also reviewed.

**Table 1:** **Summary of current and previous reported cases of mammary-type myofibroblastoma**

**Report**	**Sex/age**	**Tumor size and weight**	**Site**	**Gross description of mass**
Current case	M/50	34×28×22cm, 13Kg	Right thigh	Firm, well-circumscribed with yellow cut surface and focal myxoid change
Millo *et al*. [11]	F/46	5.3cm	Liver	Solid, non-encapsulated, fat-containing tumor
Kojima *et al*. [10]	M/79	3.5×3.5×2cm	Right seminal vesicle	Sharply demarcated, white whorled, solid mass
Wei and Zhu [15]	F/80	4×3.5×1cm	Left vulva	Firm, tan, nodular, well-circumscribed, un-encapsulated.
Arsenovic *et al*. [6]	F/40	5.5×4×4cm	Right buttock	Elastic, grayish white, well-circumscribed mass with solid, whorled cut surface
Zhang *et al*. [14]	F/40	5cm, 40g	Perianal	Well-circumscribed, encapsulated nodular mass with solid, yellow homogenous cut surface
Hox *et al*. [9]	F/45	6cm	Behind mandibular angle	Well-circumscribed, mobile and soft mass
Diwadkar and Barber [8]	F/56	3cm	Left vulva	Densely adherent to surrounding tissue
Scotti *et al*. [13]	M/36	9cm	Popliteal fossa	Nodular, whitish, well-circumscribed, un-encapsulated mass.
Mukonoweshuro *et al*. [12]	M/85	7cm, 149g	Left paratestis	Well-circumscribed, apparently encapsulated, nodular mass with solid, whorled pale-gray cut surface
Maggiani *et al*. [5]	M/37	N	Groin	Firm and well-circumscribed mass.
McMenamin and Fletcher [3] (9 cases)	M (7), F (2) (35–67)	6cm (2–13)	IA (3), AW (1), VW (1), MB (1), TA (1), BT(1), RG (1)	Mostly firm and well-circumscribed, yellow-white mass with whorled cut surface

AW, Abdominal wall; BT, Buttock; IA, Inguinal area; MB, Mid back; RG, Right groin; PA, Paratesticular area; VW, Vaginal wall.

## Case presentation

**Figure 1:**
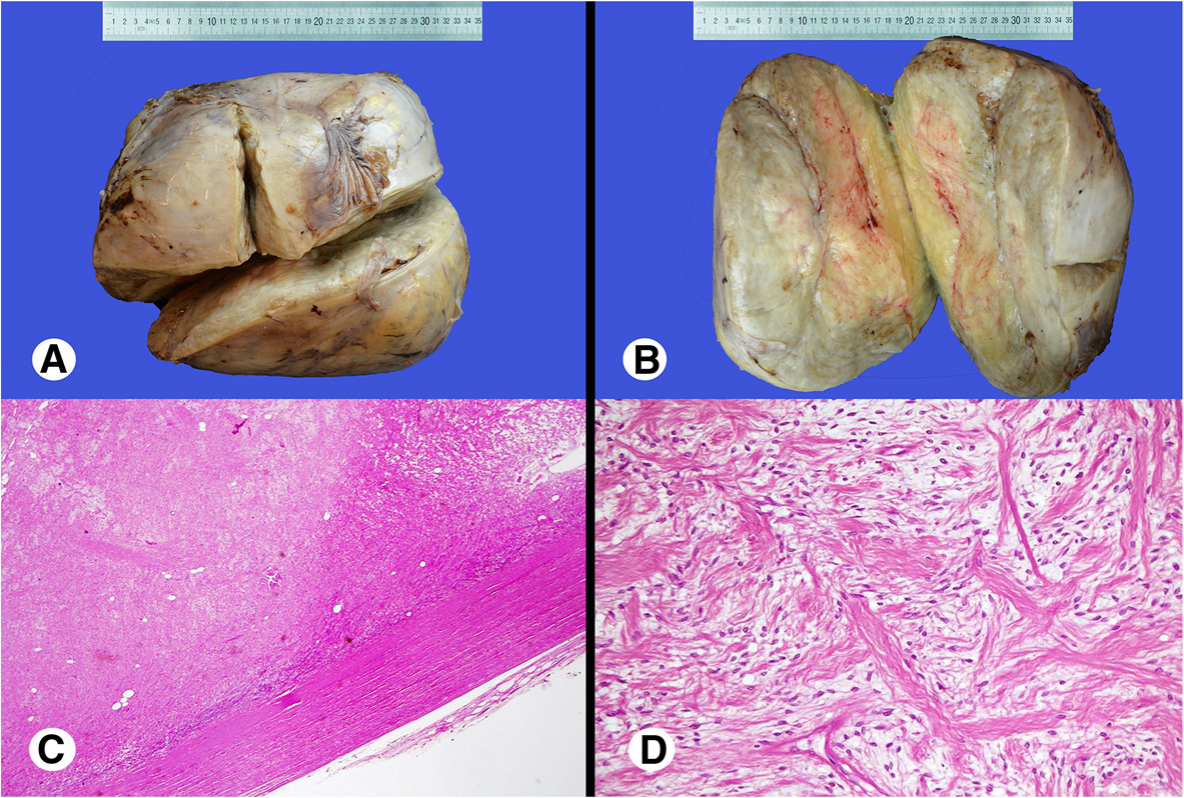
Gross and low microscopic view of the mass. **(A)** Well-circumscribed, oval to round large mass, with smooth external surface. **(B)** Sectioning reveals yellow-white, myxoid cut surface with a whorled pattern, no necrosis or hemorrhage seen. **(C)** Low microscopic field shows a well-circumscribed, un-encapsulated mass. **(D)** Bland, short, spindle-shaped cell fascicles arranged haphazardly in a collagenous stoma.

A 50-year-old Pakistani man presented to a tertiary care hospital with a long-standing history of a mass in his right thigh. His physical examination revealed a huge bulging mass on the medial side of his right thigh. No inguinal or any other local lymphadenopathy was present. A clinical impression of the soft tissue sarcoma was made and surgical excision of the mass was planned. Peroperatively, the mass was circumscribed, encapsulated and well-demarcated from underlying muscles, with no invasion of underlying tissues. Upon gross examination, the mass was well-circumscribed, firm in consistency, and measured approximately 34×28×22cm and weighed approximately 13kg (Figure 1A). The cut surface showed a pale yellow appearance, with focal myxoid change and few areas of cystic degeneration. Neither necrosis nor hemorrhage was noted (Figure 1B).

**Figure 2:**
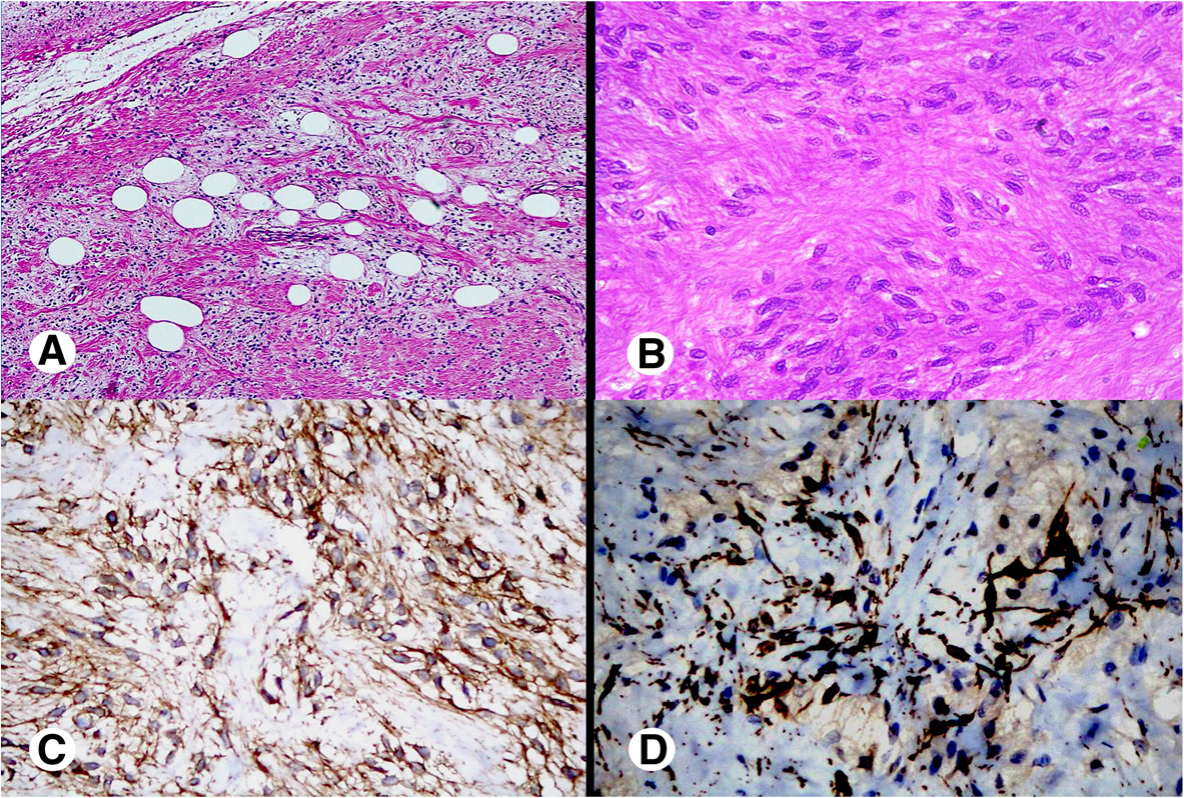
High microscopic view and immunohistochemical finding of the mass. **(A)** In this microscopic view, interrupted adipocytes are seen in a collagenous background. **(B)** High-power view shows the cells with coffee-bean shaped intranuclear grooves. Immunohistochemical stains show diffuse positivity for **(C)** CD34, and multifocal patchy positivity for **(D)** desmin.

The tumor was extensively sampled for microscopic examination. Histologically, the mass was composed of short spindle to oval-shaped cells in a haphazard arrangement of fascicles and sheets (Figure 1C,D). The cells had eosinophilic cytoplasm with indistinct cell borders and elongated nuclei with fine chromatin. Focal interspersed adipocytes are seen (Figure 2A). Nuclear grooves were noted in some cells (Figure 2B). Mitotic activity was scant and no atypical mitotic figures were found. No cytologic atypia or necrosis was noted. The stroma was composed of thick bundles of collagen with focal myxoid change and was surrounded by fatty tissue. However, the surrounding fatty tissue did not show lipoblasts or atypical adipocytes. It was revealed on immunohistochemistry that the neoplastic spindle-shaped cells showed strong positive expression for CD34 (Figure 2C), and patchy expression for desmin (Figure 2D). An immunohistochemical stain for the S-100 protein showed positive expression in the background adipocytes. However, the neoplastic cells were negative for CD10, alpha smooth muscle actin, STAT6 B-cell lymphoma 2 (Bcl 2) and epithelial membrane antigen (EMA).

## Discussion

Myofibroblastoma is a rare, benign, mesenchymal tumor of breast. An extra-mammary location is rare, and usually occurs along the embryonic milk-line, which extends from the mid-axilla to medial groin [3]. Accessory breast tissue can also be present along this embryonic line. However, several cases outside of this embryonic line have been also reported [3,6,9,13,14], which is difficult to explain on the basis of the embryonic milk-line concept.

A review of the 20 previously reported cases showed that mammary-type myofibroblastoma occurs at a relatively old age, with an average reported age of 52.5 years (range 35 to 85 years), and slight male predominance (M:F ratio = 1:0.7). Classically, these tumors are mostly slow growing, and form painless masses. The average reported tumor size was 5.5cm (range 2 to 13cm), and most of the reported tumors were small and discovered incidentally. Most cases were reported in the pelvic area: five cases in the inguinal area [3,5], three cases in the vulva and vaginal wall [3,8,15], two in the paratesticular area [3,12], and two cases in the buttock [3,6], followed by single cases reported in a seminal vesicle [10], the perianal region [14], and the suprapubic area [3]. So far, five cases have been reported outside the pelvis, including cases in the mandibular region [9], popliteal fossa [13], abdominal wall [3], liver [11], and back (Table 1) [3]. Regardless of size or location, these lesions behaved in a benign fashion, with no recurrences or metastases after surgical excision reported so far.

The mammary-type myofibroblastomas described in the literature are well-circumscribed, un-encapsulated masses, consisting of spindle-shaped cells haphazardly arranged in fascicles of various sizes, along with bands of hyalinized thick collagen in the background. Intralesional fat lobules are also seen, and intranuclear grooves have been described [1,2]. It has been revealed on immunohistochemistry that these lesions have positive expression for CD34 and desmin. We describe what, to the best of our knowledge, is the largest case of mammary-type myofibroblastoma (34cm in maximum dimension) reported so far, and is the first case reported in the medial side of the thigh.

For the proper diagnosis of mammary-type myofibroblastoma in soft tissue outside the milk-line, clinicopathologic correlation is needed. Clinically, these tumors present as slow growing, painless masses, without evidence of local lymphadenopathy, in middle-aged patients. Pathologically, careful gross examination is essential to detect the presence or absence of necrosis, a fibrotic component, hemorrhage, and so on. Extensive sectioning for microscopic examination is essential in order to rule out the presence of cellular atypia or mitosis. Positive expression for immunostaining for CD34 and desmin is helpful in reaching a correct diagnosis.

The importance of mammary-type myofibroblastoma, regardless of size or location, primarily lies in its definition as a distinctive benign neoplasm, which should not be confused with malignant soft tissue neoplasms, such as stromal sarcoma, undifferentiated pleomorphic sarcoma (malignant fibrous histiocytoma), spindle cell carcinoma, and so on [1,13].

The differential diagnosis includes both benign and malignant neoplasms. It is most often confused with spindle cell lipoma; however, other benign lesions such as angiofibroma, angiomyofibroblastoma, soft tissue perineurioma, and nodular fasciitis should also be considered and excluded [3]. Both spindle cell lipoma and mammary-type myofibroblastoma are morphologically similar benign neoplasms, composed of CD34-positive spindle cells admixed with mature adipose tissue, and both reveal loss of genetic material from the chromosome 13q14 region [5]. In mammary-type myofibroblastoma, the background stroma is more prominent and hyalinized, and the adipose tissue component is less prominent than in spindle cell lipoma [16]. Moreover, spindle cell lipoma does not stain for desmin, whereas mammary-type myofibroblastoma is always positive for this immunohistochemical marker.

Tumors with uncertain behavior, such as lipomatous hemangiopericytoma and solitary fibrous tumor, and malignant tumors, such as low-grade spindle cell liposarcoma, low-grade peripheral nerve sheath tumor, and dermatofibrosarcoma protuberans, should also be considered in the differential diagnosis. Most of these entities can be eliminated from consideration with careful attention to histologic features. Of those listed, solitary fibrous tumor histologically is perhaps the closest mimic of mammary-type myofibroblastoma. Solitary fibrous tumor has a more prominent hemangiopericytomatous vasculature, and is also immunohistochmically positive for signal transducer and activator of transcription 6 (STAT6) protein [17].

## Conclusions

Mammary-type myofibroblastoma of the soft tissue is a benign, soft tissue neoplasm, and no malignant behavior and/or recurrence after surgical resection has been described in the cases reported so far. As an extremely rare tumor, the correct diagnosis and prompt management is important, and requires careful clinical and pathological workup and extensive sectioning after surgical excision to demonstrate the presence or absence of necrosis, mitosis, or nuclear atypia, and thus rule out a malignant neoplasm.

## Consent

Written informed consent was obtained from the patient for publication of this case report and accompanying images. A copy of the written consent is available for review by the Editor-in-Chief of this journal.
